# Optical coherence tomography angiography findings in patients with branch retinal vein occlusion treated with Anti-VEGF

**DOI:** 10.5935/0004-2749.20200017

**Published:** 2020

**Authors:** Emine Ciloglu, Neşe Çetin Dogan

**Affiliations:** 1 Department of Ophthalmology, Adana City Training and Research Hospital, Adana, Turkey

**Keywords:** Retinal vein occlusion, Tomography, optical coherence, Endothelial growth factors/antagonists & inhibitors, Oclusão da veia retiniana, Tomografia de coerência óptica, Fatores de crescimento endotelial/antagonistas & inibidores

## Abstract

**Purpose:**

To investigate retinal microvasculature changes in patients treated with anti-VEGF for macular edema secondary to branch retinal vein occlusion.

**Methods:**

We examined 38 eyes of 19 patients for the study. We measured superficial and deep capillary plexus vessel densities (%), foveal avascular zone areas (mm^2^), and central macular thicknesses.

**Results:**

Parafoveal superficial and deep capillary plexus values were significantly lower in eyes with branch retinal vein occlusion than in fellow eyes (p<0.001). We found a significant increase in parafoveal deep capillary plexus values after the anti-VEGF treatment (p=0.032). The mean foveal avascular zone was larger in eyes with branch retinal vein occlusion than in control eyes (p<0.001). The mean central macular thickness was significantly higher in eyes with branch retinal vein occlusion than in controls, and we observed a significant decrease in central macular thickness after anti-VEGF treatment (<0.001). In addition, the cystic structures in the deep capillary plexus regressed.

**Conclusion:**

Optical coherence tomography angiography enables qualitative and quantitative evaluations during follow-up of patients treated for branch retinal vein occlusion.

## Abstract (pt)

**Objetivo:**

Investigar as alterações na microvasculatura da retina em pacientes tratados com anti-VEGF para edema macular secundário à oclusão de ramo da veia retiniana.

**Mé todos:**

Foram examinados 38 olhos de 19 pacientes para o estudo. Medimos as densidades dos vasos do plexo capilar superficial e profunda (%), áreas da zona avascular foveal (mm^2^) e espessura macular central.

**Resultados:**

Os valores do plexo capilar superficial e profundo parafoveal foram significativamente menores nos olhos com oclusão de ramo da veia retiniana do que nos outros olhos (p<0,001). Encontramos um aumento significativo nos valores de plexo capilar profundo parafoveal após o tratamento com anti-VEGF (p=0,032). A zona avascular foveal média foi maior nos olhos com oclusão de ramo da veia retiniana do que nos olhos controle (p<0,001). A espessura macular central média foi significativamente maior nos olhos com oclusão de ramo da veia retiniana do que nos controles, e observamos uma diminuição significativa na espessura macular central após o tratamento com anti-VEGF (< 0,001). Além disso, as estruturas císticas no plexo capilar profundo regrediram.

**Conclusão:**

A angiotomografia de coerência óptica permite avaliações qualitativas e quantitativas durante o acompanhamento de pacientes tratados por oclusão de ramo da veia retiniana.

## INTRODUCTION

Retinal vein occlusion (RVO) is the most common cause of retinal vascular disease after diabetic retinopathy. The most important cause of visual impairment in RVO is macular edema^(1)^.

Fundus fluorescein angiography (FFA) and optical coherence tomography (OCT) devices can be used to diagnose RVO and to monitor clinical findings and treatment response. FFA detects neovascularizations and nonperfused areas in the posterior pole and peripheral retina. SD-OCT is the most useful device for identifying macular edema. Moreover, it allows the evaluation of individual retinal layers, and it is used to evaluate macular edema changes after treatment^(2-5)^.

Optical coherence tomography angiography (OCTA) is a new noninvasive imaging technique that does not req uire dye injection. OCTA enables high resolution and rapid imaging of the blood stream in various layers of the retina and generates a three-dimensional image reflecting these vascular layers that allows quantitative measurements of the vascular network. OCTA facilitates individual analyses of distinct vascular layers of the retina, namely, the superficial and deep capillary plexuses^(6)^.

As imaging with FFA is limited to the superficial capillary plexus (SCP), OCTA is superior to FFA for diagnosing diseases that affect the deep capillary plexus (DCP)^(7)^.

Studies have shown that the most important vascular changes occur in the DCP, and decreased perfusion and DCP ischemia are important for visual prognoses^(8,9)^. Moreover, the “en face” imaging mode of OCTA enables acquisition of the image of a normal foveal avascular zone (FAZ) like in FFA.

In OCTA, the shape and size of the FAZ can be assessed, and FAZ changes can be followed by revealing deteriorations in the perifoveal microvascular stream reaching the central macula^(10,11)^.

Studies evaluating retinal vascular diseases, including age-related macular degeneration, macular telangiectasia, diabetic retinopathy, and RVO with OCTA, exist^(12-16)^.

Coscas et al. detected abnormalities of retinal capillaries in the networks of both the SCP and the DCP, with those being more pronounced in the DCP^(14)^.

Capillary dropout, deterioration of perifoveal arcade, and enlargement of FAZ have been reported in patients with RVO^(14,17-20)^.

For this study, we evaluated the effect of antivascular endothelial growth factor (anti-VEGF) treatment in eyes with branch retinal vein occlusions (BRVOs) using OCTA by comparing the baseline (eye with BRVO before treatment) and after treatment findings (same eye after treatment) with the contralateral eye findings. We assessed vessel densities of the SCP and DCP, cystic structures, and FAZ areas by OCTA in patients who underwent anti-VEGF treatment because of macular edema secondary to BRVO.

## METHODS

We collected data retrospectively from the medical records of the patients who were treated and followed for macular edema secondary to BRVO in our retina cli nic between March 2017 and February 2018.

We performed this clinical study in agreement with the tenets of the Declaration of Helsinki with the approval of the local ethics committee. We obtained individual informed consents prior to obtaining the patients’ data.

We included data from patients who had received three loading doses of anti-VEGF therapy (within three-month periods) for macular edema secondary to BRVO. Macular edemas were treated by intravitreal injections of 2 mg aflibercept (EYLEA; Regeneron Pharmaceuticals, Tarrytown, NY, USA, and Bayer, Berlin, Germany).

We excluded patients with macular edema due to other ocular diseases (such as central RVO, diabetic retinopathy, and stage 4 hypertensive retinopathy diagnosed with fundoscopic examination and SD-OCT); those with histories of cigarette use, ocular surgery, laser or intravitreal injections due to macular edema, age-re la ted macular degeneration, epiretinal membrane, vitreous hemorrhage, BRVO with severe hemorrhage, and uveitis; and those with high myopia (>-6 diopters) or with significant media opacities. We also excluded data from patients diagnosed without OCTA or from those with poor quality images (defined as scan quality <6/10 or presence of significant artifacts).

BRVO diagnoses were based on medical and ophthalmic histories and on full ophthalmic examinations. All patients underwent comprehensive ophthalmic examinations, including best corrected visual acuity (BCVA), and slit-lamp biomicroscopic and fundus examinations. FFA and SD-OCT were used for retinal assessments. The BCVA was measured using a Snellen chart, and the decimal visual acuity was converted to logarithm of the minimal angle of resolution (logMAR) units.

An SD-OCT apparatus (Retina Scan RS 3000 Advance, Nidek, Gamagori, Japan) was used to measure the central macular thickness. We captured the OCT scans using the macula line 12-mm horizontal scan. The scans consisted of 1,024 A-scans with high definition. Each image consisted of 120 averaged B-scans with a 4-µm resolution.

All OCTA images were obtained using the AngioVue system (Optovue RTVue XR Avanti; Optovue, Fremont, California). This instrument has an A-scan rate of 70,000 scans per second and uses a light source centered on 840 nm and a bandwidth of 45 nm.

We evaluated the 3 × 3-mm OCT angiograms for the following characteristics: FAZ areas (mm^2^), foveal and parafoveal vessel densities (%) in the SCP and DCP, and capillary nonperfusion. The AngioVue software provides an automated FAZ boundary detection applied on a retina slab (ILM to DPL +10 µm) and can be reviewed on the “en face” screen under “FAZ” measurements.

The superficial and deep retinal vascular networks were automatically calculated using built-in software.

The “en face” image was then automatically segmented with an inner boundary 3 µm beneath the internal limiting membrane and an outer boundary 15 µm beneath the inner plexiform layer (IPL) to obtain SCP images. The segmentation was carried out with an inner boundary 15 µm beneath the IPL and an outer boundary 70 µm beneath the IPL to obtain DCP images.

A projection artifact removal algorithm is available with our version of AngioVue software. We evaluated images with high scan quality (score >6). In patients with multiple available OCTA images, the one with the highest scan quality was used for analysis. Segmentation errors, if present, were corrected manually for each patient.

Parafoveal vessel densities (VDs) were calculated for the ring-shaped area between 1 and 2.5 mm from the center of the fovea. Parafoveal VDs were defined as the percentage of the total area occupied by vessels and microvasculature and were quantified in the SCP and DCP. We used the AngioVue Analytics software to calculate VDs and extract a binary image of the blood vessels from the gray-scale OCTA image and then calculate the percentage of pixels with a flow signal greater than the threshold in the defined region.

The treatment regimen started with three monthly injections. After this loading phase, the injections were continued in the presence of macular edema.

The measurements were conducted before and at least one month after the loading dose of anti-VEGF. We compared the measurements of BRVO eyes before treatment with those of healthy fellow eyes and with the values measured after treatment.

### Statistical analyses

We performed statistical analyses using the Statistical Package for the Social Sciences software version 20.0 (IBM, Chicago, IL, USA). We assessed the appropriateness of the calculations for normal distribution using the Kolmogorov-Smirnov test. For parametric comparisons, we applied the Student t-test for two independent groups and the Mann-Whitney U test for variables without normal distributions. We adopted a 5% level of significance and considered results with a p-value <0.05 as significant.

## RESULTS

We included data from 38 eyes of 19 patients (12 men and seven women) in the study. The mean age of the patients was 59.8 ± 10.4 years.

The mean disease duration at the time the patients applied to our clinic was 1.9 ± 1.5 months. The mean time between the initial examination and first intravitreal injection was 2.2 ± 1.3 months. Eight patients had a history of hypertension, two had glaucoma, and four had dyslipidemia.

Before treatment, the mean BCVAs were 0.52 ± 0.35 (logMAR) in the BRVO eye and 0.10 ± 0.15 (logMAR) in the fellow eye. The mean visual acuity increased significantly to 0.15 ± 0.20 in the BRVO eyes after three doses of anti-VEGF treatment (p<0.001)

### OCTA findings

FAZ was larger in the BRVO eyes (0.705 mm^2^ in eyes with BRVO and 0.41 mm^2^ in control eyes; p<0.001).

Comparisons of the BRVO and control eye measurements showed that the parafoveal VDs at the SCP and DCP were significantly lower in the BRVO eyes than in the control eyes (44.83% ± 5.23%, 49.7% ± 4.32%; p<0.001, and 45.6% ± 5.41%, 51.8% ± 5.38%; p<0.001; respectively). The VDs were low in all quadrants of the parafoveal region (Table 1).

**Table 1:** Comparison of OCTA parameters between groups

	BRVO	Fellow eyes	p-value
CMT	351 ± 55	202 ± 15	0.004
FAZ area mm^2^	0.70 ± 0.24	0.41 ± 0.18	<0.001
Foveal VD (%) Superficial	30.56 ± 6.84	31.04 ± 5.43	0.735
Deep	29.41 ± 2.51	28.52 ± 9.93	0.639
Parafoveal Superficial	44.83 ± 5.23	49.7 ± 4.32	<0.001
Deep	45.6 ± 5.41	51.8 ± 5.38	<0.001

CMT= central macular thickness; FAZ= foveal avascular zone; VD= vessel density; BRVO= branch retinal vein occlusion

We did not find differences in vessel density values of foveal SCP and DCP when comparing BRVO and contralateral eyes.

We found a significant increase in parafoveal DCP vessel density (48.46% ± 6.01%; p=0.032) and a significant FAZ area reduction after anti-VEGF treatment (p<0.001).

Although central macular thickness (CMT) values were significantly higher in the eyes with BRVO (351 ± 55 µm) than in contralateral unaffected eyes, they were significantly reduced after treatment (196.1 ± 33 µm; Table 2).

**Table 2:** OCTA parameters in patients with BRVO after treatment

BRVO	Before treatment	After treatment	p-value
CMT	351 ± 55	196 ± 33	<0.001
FAZ area mm^2^	0.70 ± 0.24	0.54 ± 0.18	0.001
Foveal VD (%) Superficial	30.56 ± 6.84	30.82 ± 4.45	0.665
Deep	29.41 ± 2.51	29.52 ± 2.49	0.772
Parafoveal Superficial	44.83 ± 5.23	45.75 ± 4.46	0.445
Deep	45.6 ± 5.41	48.46 ± 6.01	0.032

CMT= central macular thickness; FAZ= foveal avascular zone; VD= vessel density; BRVO= branch retinal vein occlusion.

In the presence of macular ischemia, we frequently found perifoveal arcade disruptions.

In the DCP, we observed rarefaction of vessel density with telangiectatic appearance of retinal vessels, particularly in the areas of macular edema.

**Figure 1:**
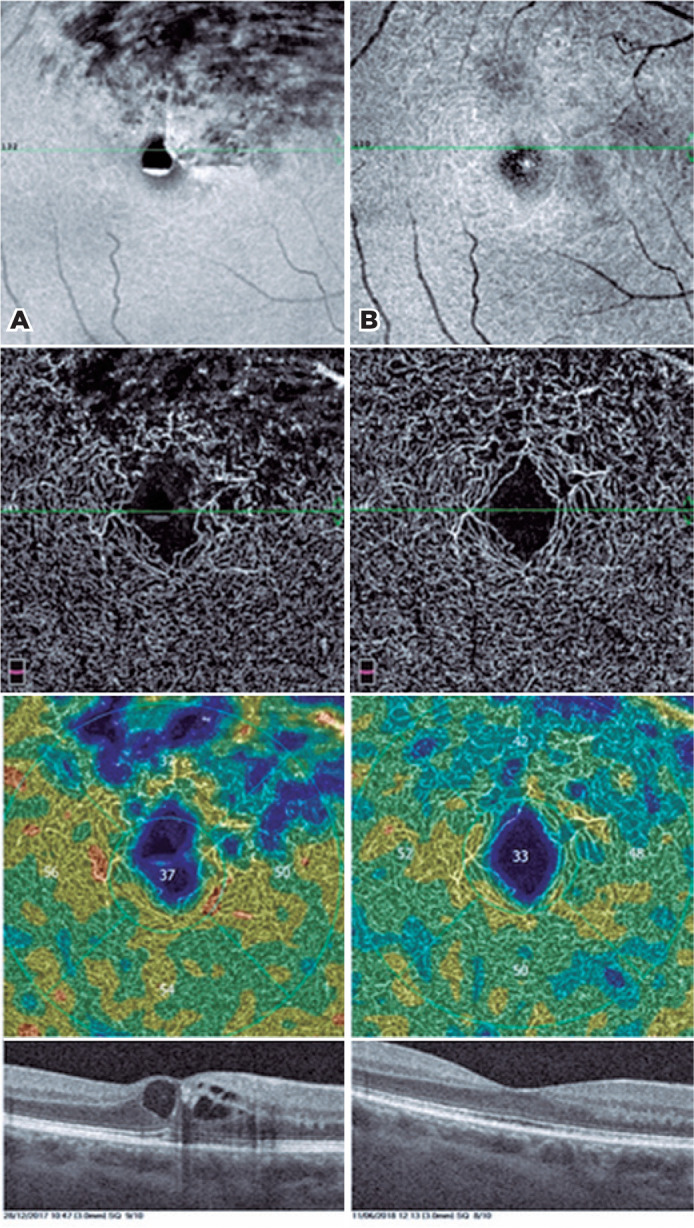
En face OCT and OCTA images of a patient before (Panel A) and after (Panel B) treatment. En face OCTA images at the level of the deep retinal plexus (Panel A, top) showing central hyporeflective spaces due to intraretinal cysts, retinal hemorrhages, and retinal edema. Macular vascular map (Panel A, bottom) showing nonperfused areas on the superior quadrant. Corresponding OCTA frame at the level of the deep plexus (Panel A, middle) showing telangiectatic and rarified vessels and flow-raid spaces. En face OCTA images at the level of deep retinal plexus (Panel B, top) complete recovery of retinal structure. Almost normal vessel caliber and recovery of perifoveal vascular arcade (Panel B, middle). Reduced nonperfusion areas and increased vessel density (Panel B, bottom).

**Figure 2:**
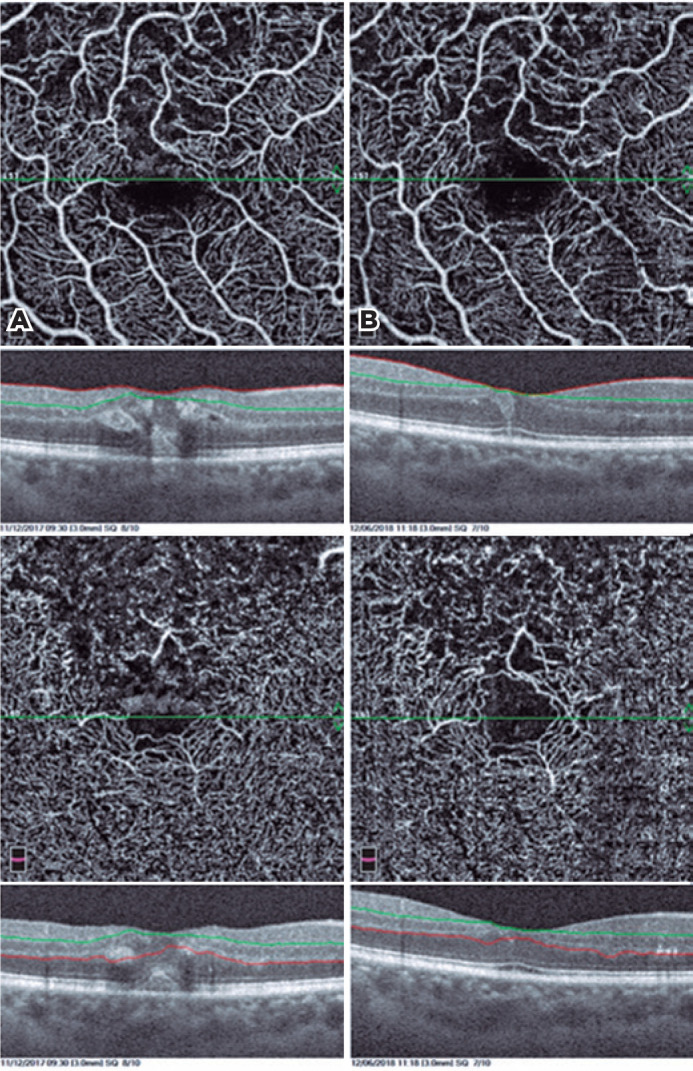
OCTA images of a patient before treatment (Panel A) and after treatment (Panel B). Superficial capillary plexus (SCP) showing disruption of perifoveal vascular arcade (Panel A, top) and shows complete absence of edema and an intact perifoveal capillary arcade after treatment (Panel B, top). Deep capillary plexus (DCP) showing dilatation of the superior macular capillaries with hypersignal and foveal avascular zone (FAZ) disruption (Panel A, bottom) and showing recovery of perifoveal vascular arcade after treatment (Panel B, bottom).

**Figure 3:**
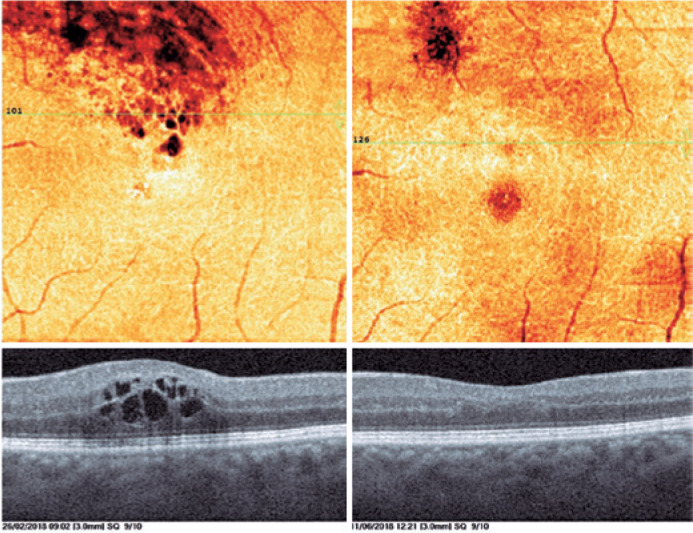
En face color images of OCTA of a patient showing the regression of hemorrhages and cysts after treatment.

**Figure 4:**
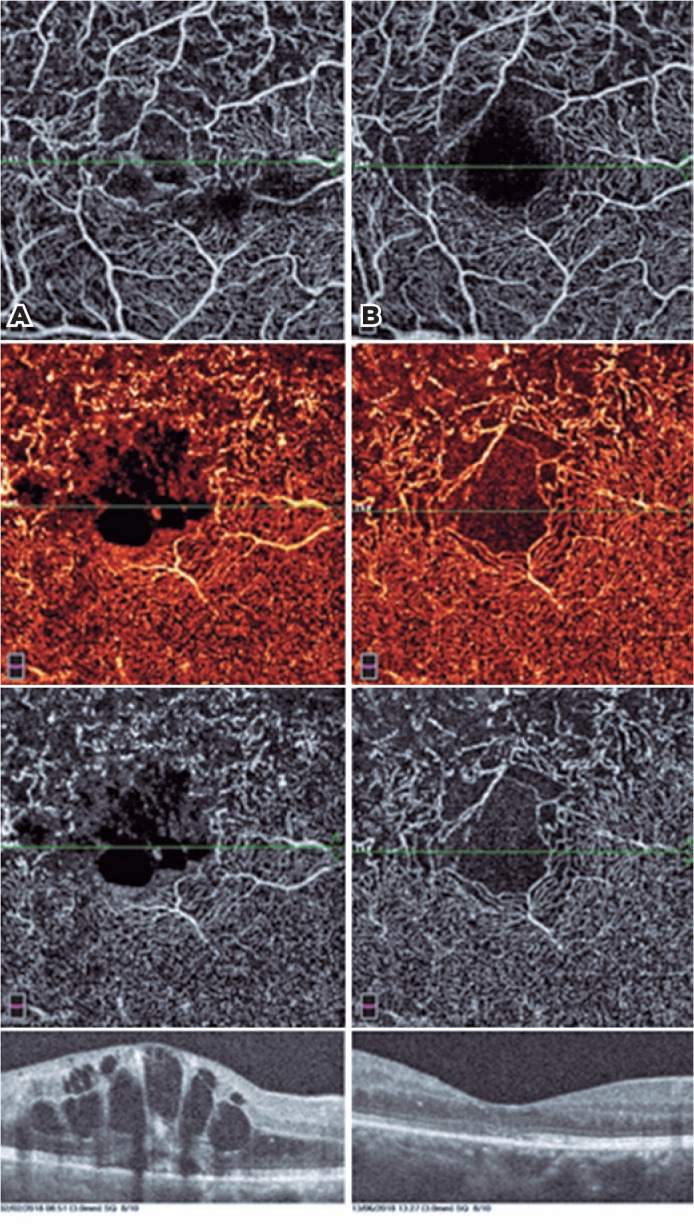
OCTA images of patients before (Panel A) and after (Panel B) treatment. Superficial capillary plexus (SCP) showing disruption of FAZ and vessel dilatations (Panel A, top). After treatment, the SCP shows recovery of the perifoveal vascular arcade and normalization of the capillary network (Panel B, top). The color images of en face OCTA of the deep capillary plexus (DCP) showing cystoid spaces before treatment and regressions after treatment. The DCP shows vascular dilatation and telangiectatic vessels (Panel A, bottom) before the treatment and recovery of the perifoveal anastomotic arcade after the treatment (Panel B, bottom).

In addition, the cystic structures, especially those in the deep vascular layer, were stretched to a great extent in OCTA images. The perifoveal capillary arcade improved remarkably after the treatment (Figures 1-4).

## DISCUSSION

In this study, we investigated retinal superficial and deep VDs and FAZ areas using OCTA in patients with BRVO complicated by macular edema at baseline and after intravitreal anti-VEGF treatments.

When compared with the unaffected eyes of the patients before treatment, we found the FAZ areas in BRVO eyes to be significantly larger than those in the fellow eyes.

In the BRVO eyes, we also noted a decrease in the vascular densities at the parafoveal SCP and DCP compared to those in treated and contralateral eyes, and the reductions were more pronounced in the vascular occlusion quadrants than in other sites. The results of our study confirm those of Samara et al., who reported a reduction in vascular densities in both the SCP and DCP correlating FAZ areas, VDs, and visual acuities^(21)^.

Superficial and deep foveal VDs were similar between eyes with BRVO and fellow eyes. This may be because ischemic changes do not affect the foveal vessel density because of the presence of a FAZ with a low vein density in the region^(5,17,22)^. Similarly, Kang et al. reported similar superficial and deep foveal VDs among eyes with RVO, fellow eyes, and control eyes^(5)^.

Studies have shown capillary network abnormalities in the deep capillary microvasculature in patients with RVO^(5,17,20,23,24)^. Paques et al. showed major veins in the superficial layer connected directly to the deep layer through transverse venules. This may elevate the hydrostatic pressure in the deep circulation, leading to decreased perfusion of deep retinal structures. In addition, the superficial layer is connected with retinal arterioles supported by a high perfusion pressure, and this may explain why the SCP is better preserved than the DCP in RVO eyes^(25)^.

In cases of BRVO, Kashani et al. reported that OCTA findings are consistent with clinical, anatomic, and fluorescein angiographic findings such as decreased vascular perfusion, vascular dilatation, collateral vessels, and intraretinal edema^(19)^. Suzuki et al. found that OCTA can be used to visualize the microvascular abnormalities in macular edema associated with BRVO as well as FFA^(20)^.

In our study, we evaluated our patients with both FFA and OCTA before and after treatment, and we were able to evaluate the microvascular changes in both the SCP and DCP with OCTA. We determined recovery of perifoveal vascular arcade, regression of cysts, improvement in capillary network, and reductions in nonperfusion areas.

In patients with BRVO, macular edema is the most prevalent cause of decreased visual acuity. Macular edema is caused by the release of substances that enhance vascular permeability, such as VEGF, which is produced in the retina because of the deterioration of the tight junctions between capillary endothelial cells and the adhesions between the vitreous and the retina damaging the blood-retinal barrier^(26)^. Anti-VEGF drugs are used frequently in the treatment of macular edema due to BRVO.

A slight decrease in average macular vessel density, despite the resolution of macular edema, has been shown in patients with RVO and macular edema treated with intravitreal injections of anti-VEGF or with dexamethasone^(27,28)^. According to Sellam et al., this reduction probably reflects the extension of retinal nonperfusion and conversion from nonischemic to ischemic RVO over time in patients with CRVO and BRVO, and in their study after treatment, vascular density (measured by AngioAnalytics) improved in only 36% of patients^(27)^. We found no significant differences in vascular density before and after treatment in BRVO eyes.

The capillaries could be displaced at the periphery of the cysts as mentioned by Couturier et al.^(13)^. An increa se in vascular density may be caused by the regression of the cysts after treatment. In our study, we found an increase in parafoveal vessel density after treatment. This is probably due to the cysts being stretched and the emergence of the underlying intact capillary bed.

Campochiaro et al. demonstrated that an aggressive VEGF blockage may reduce the progression of retinal nonperfusion, but cannot totally prevent it^(29)^. Monthly anti-VEGF injections may potentially improve outcomes because of a secondary reduction in ischemia progression demonstrated by Mir et al.^(30)^.

In our study, we treated patients with anti-VEGF injections monthly, and the anti-VEGF treatment significantly improved BCVAs and decreased CMTs. A strength of our study is that our patients consisted of naive patients who had not been treated before and who applied to our clinic during the acute phase of the disease.

We are aware of the limitations of our study, with its retrospective design and small sample size being the most important. In addition, the presence of macular edema may lead to segmentation and vessel density ana lysis errors.

In conclusion, OCTA showed significant regression of macular edema, reduced capillary disruption and cysts, and perifoveal vascular arcade improvements. Thus, OCTA enables qualitative and quantitative evaluations during the follow-up of patients treated for BRVO.
